# Hygienic practice during complementary feeding and associated factors among mothers of children aged 6–24 months in Borecha Woreda, southwestern Ethiopia: a community-based cross-sectional study

**DOI:** 10.3389/fped.2024.1321558

**Published:** 2024-10-07

**Authors:** Rabira Tariku Birdida, Erean Shigign Malka, Efrem Negash Kush, Fikadu Tolesa Alemu

**Affiliations:** ^1^ Department of Public Health, Mettu University College of Health Sciences, Mettu, Ethiopia; ^2^ Department of Public Health, College of Medicine and Health Sciences, Salale University, Fitche, Ethiopia; ^3^ Department of Midwifery, College of Medicine and Health Sciences, Salale University, Fitche, Ethiopia

**Keywords:** hygienic complementary feeding practice, mothers of children aged 6–24 months, Borecha Woreda, Southwestern Ethiopia, children aged 6–24 months

## Abstract

**Introduction:**

Complementary feeding is a critical transitional phase in the life of an infant, and complementary foods should be prepared, stored, and fed hygienically, with clean hands, dishes, and utensils to prevent diseases. However, the prevalence and risk factors associated with hygienic complementary feeding practice have not been well addressed in Ethiopia, especially in the study area.

**Objective:**

This study aimed to assess hygienic practices and associated factors during complementary feeding among mothers with children aged 6–24 months in Borecha Woreda, Buno Bedele Zone, southwestern Ethiopia, in 2022.

**Methods:**

A community-based cross-sectional study was conducted using a multistage sampling technique. The study was conducted on 536 mothers of children aged 6–24 months in Borecha Woreda from 1 March to 1 April 2022. Data were collected using an interviewer-administered questionnaire. The data were coded, cleaned, edited, and entered into Epi-Data version 4.6 and then exported to SPSS version 20.0 for further analysis. Both bivariate and multivariable logistic regression models were fitted. Moreover, crude (for bivariate) and adjusted (multivariable) odds ratios (AORs) with 95% confidence intervals (CIs) were computed to assess the association between independent factors and outcome variables. A *p*-value of <0.05 was considered statistically significant.

**Result:**

Out of 536 respondents, 350 (65.3%) exhibited poor hygienic practices during complementary feeding. The risk of poor hygienic practices was about two times higher among women over 30 than those under 25 (AOR: 1.8; 95% CI: 1.11–2.90). Mothers whose husbands had primary education or higher had a 50% reduced risk (AOR: 0.50; 95% CI 0.31–0.83) of poor hygienic care practices during complementary feeding compared to their counterparts. The odds of poor hygiene practice among mothers with poor knowledge of hygienic care were 2.71 times higher than those with good knowledge (AOR: 2.71; 95% CI: 1.60–4.61).

**Conclusion:**

The prevalence of poor hygienic practices during the preparation and handling of complementary foods among mothers in Borecha Woreda is high compared to other studies. Therefore, improving hygiene practices during complementary food preparation requires the involvement and responsibility of policymakers, the community, and families.

## Introduction

Food hygiene is a scientific method of handling, preparing, and storing food to reduce the risk of contracting a foodborne illness. Food can serve as a growth and reproduction medium for pathogens like bacteria, viruses, molds, and fungi, which can cause infections that can cause illness or death in people or other animals (1).

According to World Health Organization (WHO) definitions, complementary feeding (CF) is the process that begins when breast milk alone is insufficient and additional foods and liquids are introduced to meet the nutritional requirements alongside breast milk (2). It is a transition from exclusive breastfeeding to a family diet and is a critical period during which poor hygiene practices related to CF can contribute significantly to the increase in gastrointestinal and respiratory infections in many young children (3).

The WHO and United Nations International Children's Emergency Fund (UNICEF) propose that complementary foods, which begin after 6 months of age, be prepared, stored, and fed with clean hands and clean utensils rather than bottles and teats. In addition, cooked food should be carefully stored at the proper temperature. Prepared food needs to be kept cold, ideally in a refrigerator (below 5°C), or kept hot (over 60°C). The main cause of contamination in complementary meals is the prolonged storage of prepared food at room temperature for more than one feeding (2).

Proper and hygienic adherence to CF is important for achieving healthy and sustained growth and survival of young children in their early years. CF has the potential to prevent about 6% of all deaths under the age of 5, especially in developing countries (2, 4). Complementary foods provided in low-income countries may pose microbiological safety risks and can lead to foodborne pathogens. These microbiologically contaminated diets are particularly harmful to children under 2 years, as they have immature immune systems and are easily susceptible to infections from enteric pathogens (5, 6).

In various regions across the globe, the prevalence of hand-washing practices with soap at critical moments ranges from 2% to 35% (7). In many parts of developing countries, foodborne diseases and their prevention are poorly understood and have received less attention. Even public health authorities are not fully cognizant of the potentially enormous health and economic burden these foodborne diseases impose on their societies (8).

According to the Ethiopian Mini Demographic and Health Survey 2019 report, the prevalence of infant mortality caused by diseases from preventable bacterial pathogen contamination was 43% (9). In addition, the prevalence of hygienic practices during complementary feeding among children aged 6–24 months in Tegedie Woreda, northwest Ethiopia was found to be 33.6% (3). Recent evidence from the Bahir Dar area, north Ethiopia, indicated that about 62% of study participants exhibited poor hygienic practices during complementary feeding (6).

In developing nations, contamination of complementary foods is prevalent due to factors such as contaminated water, poor personal hygiene, inadequate cleaning of utensils, improper food storage after preparation, limited knowledge of safe food handling, poor environmental sanitation, and inadequate social services related to bathrooms, kitchens, and sewage systems (8).

Most under-5 deaths from diarrhea occur in children living in developing countries (10). Up to 70% of childhood diarrhea episodes in these regions are attributed to poor food hygiene practices. Factors such as inadequate personal hygiene, unsafe water supplies, and poor sanitation contribute to approximately 88% of childhood mortality (2, 11). A study conducted in rural Bangladesh demonstrated that children whose caregivers washed at least one hand with soap before food preparation experienced significantly less diarrhea (3.7%) compared to children in households (HHs) where caregivers did not practice hand washing (12.5%) (12).

Consistently promoting caregivers to wash their hands with soap before preparing food and feeding the child could reduce illness and pathogen transmission, potentially improving child growth. Understanding the risk associated with hygiene practices during complementary feeding is crucial for preventing and controlling foodborne infections in children aged 6–24 months, and enhancing food hygiene practices may play a significant role in reducing child morbidity and mortality (4, 13).

Despite the existing challenges, the prevalence and risk factors associated with hygienic complementary feeding practices remain inadequately addressed in Ethiopia, particularly in the study area. Therefore, this study assessed the prevalence of hygienic practices and the factors associated with hygiene during complementary feeding among women in Borecha Woreda, Oromia, Western Ethiopia.

## Methods

### Study area

The research was conducted in Borecha Woreda, situated in the southwestern region of Ethiopia within the Buno Bedele Zone of the Oromia Regional State. This region is approximately 36 km from Bedele town and 430 km from Addis Ababa, the capital city of Ethiopia. Borecha Woreda comprises 33 kebeles, including 2 urban and 31 rural areas. According to the reports from the Borecha Woreda Health Office in 2021, the total population residing within Borecha Woreda was 107,490, consisting of 52,670 males and 54,820 females. Borecha Woreda contained 5,159 households, with an estimated 20,057 mothers engaged in breastfeeding. In addition, there were 4,600 households with children aged between 6 and 23 months. Moreover, Borecha Woreda was served by 5 governmental health centers and 13 private health institutions providing healthcare services (source: Borecha Woreda Health Office record of 2021).

### Study design and study period

A community-based cross-sectional study was conducted from 1 March to 1 April 2022.

### Source population

The source population for the study consisted of all mothers of children aged 6–24 months living in Borecha Woreda.

### Study population

The source population consisted of all mothers/caregivers with children aged 6–24 months living in the randomly selected kebeles in Borecha Woreda during the data collection period.

### Inclusion and exclusion criteria

#### Inclusion criteria

Mothers/caregivers who were permanent residents (who had lived in Borecha Woreda for at least 6 months) and had infants or young children aged 6–24 months were included in the study.

#### Exclusion criteria

Mothers/caregivers with children aged 6–24 months who were seriously ill and unable to respond at the time of data collection were excluded.

#### Sample size determination

The sample size (*n*) for prevalence was calculated with the assumptions of a single population proportion, an estimated proportion of 33.6% from a previous study conducted in Tegedie Woreda, northwest Ethiopia (3), *Z*_a/2_ = 1.96, which is the critical point for the standard normal tabulated value at a 95% confidence interval, and a margin of error (*d*) of 5%.

Thus, *p* = 0.336, 1–*p* = 1–0.336 = 0.664.

$$n=\frac{Z^{2}\alpha /2\;\times \;p\;(1-p)\;}{\;w^{2}}$$

$$\frac{{\left(1.96\right)}^{2}\;\times \;0.336\;(0.664)\;}{\;{\left(0.05\right)}^{2}}=343$$

where

• *n* = sample size,
• *p* = proportion, and
• *W* = maximum allowable error (margin of error) = 5%.

The final sample size, with a 5% non-response rate and considering a 1.5 design effect, was 539.

The sample size for the second objective was calculated using Open-Epi 3.01 version software (Table 1).

**Table 1:** Sample size calculation using potential variables for the prevalence of mother's hygienic practices and associated factors during complementary feeding from the previous studies.

Exposure variables	Proportion of unexposed (%)	AOR	Sample size	Non-response rate	Final sample size	Reference
Urban residence	36.5	7.02	304	16	320	(4)
Separate raw and cooked foods	33.9	5.87	54	3	57	(4)
Dishwashing facility	20.45	5.7	58	3	61	(4)

AOR, adjusted odds ratio.

The largest sample size was obtained using the single population proportion formula, which was specifically calculated to evaluate the prevalence of maternal hygiene practices during the complementary feeding period, yielding a total of 539. As a result, the necessary final sample size included 539 mothers with children aged between 6 and 24 months.

#### Sampling technique

A multistage sampling method was employed to select participants for the study. Of the 33 kebeles in Borecha Woreda, 10 (30%) were selected through a simple random sampling method. The sample size was proportionally allocated to each chosen kebele according to the population size of mothers of children aged 6–24 months residing there. A roster of these mothers, obtained from health posts in each kebele, served as the sampling frame. Following the proportional allocation, a systematic random sampling method was utilized to select the study participants from the family folders of mothers of children aged 6–24 months. The sampling interval (*K*th value) was calculated by dividing the total number of mothers of children aged 6–24 months in each kebele by the sample size designated to that kebele.

**Figure 1:**
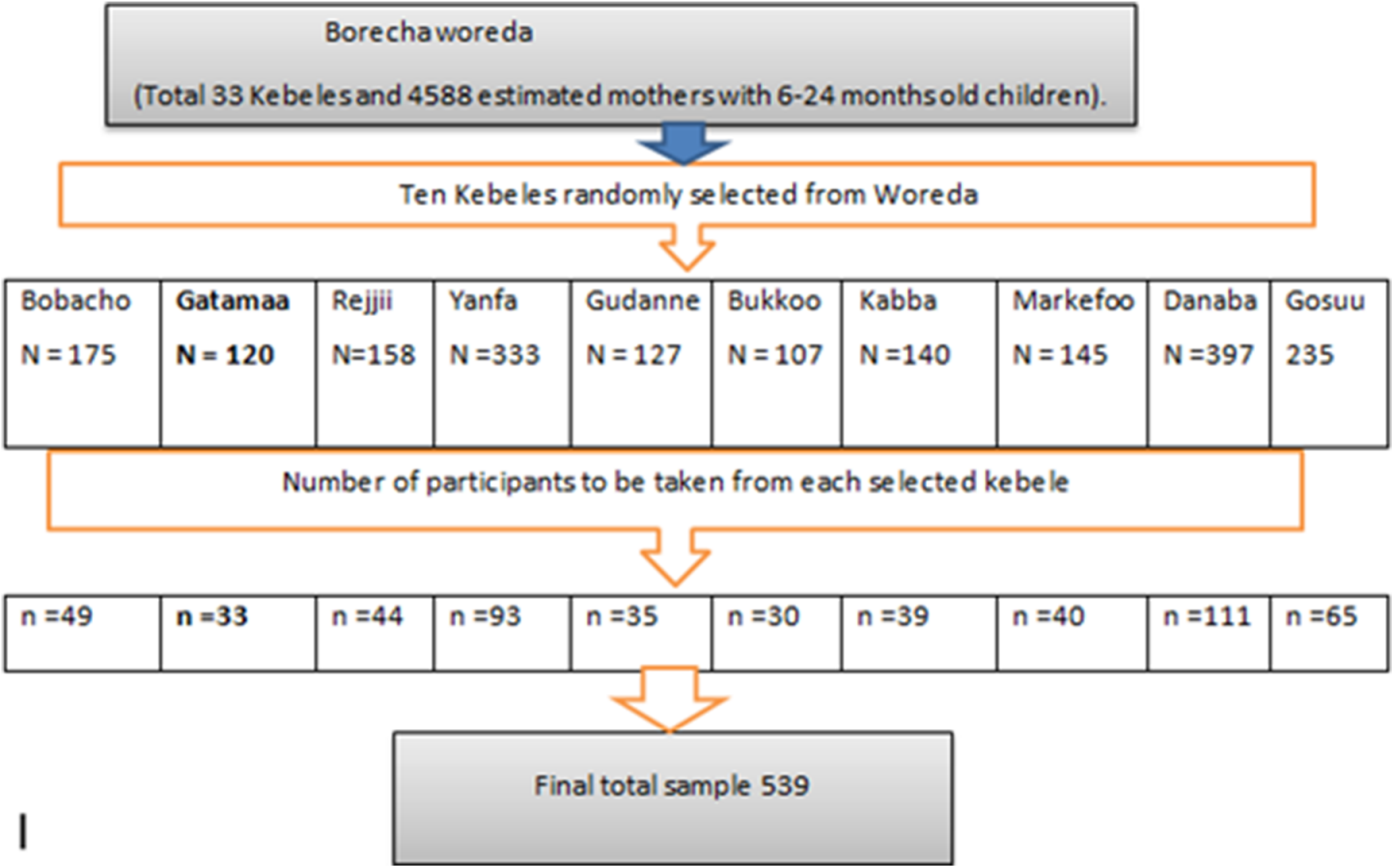
Diagram illustrating the sampling method employed for the hygienic practices during complementary feeding among caregivers in Borecha Woreda, 2022.

Here, *n* denotes the sample size and *N* indicates the total number of mothers with children aged 6–24 months in randomly selected kebeles (Figure 1).

### Study variables

#### Dependent variable

Hygienic practices during complementary feeding served as the dependent variable.

#### Independent variables

Independent variables included maternal factors, such as age, education, knowledge, occupation, marital status, and place of delivery; household and social factors, including religion, ethnicity, paternal education, paternal occupation, family size, monthly income, type of latrine, and source of drinking water; child factors, such as age and sex of the child, growth monitoring follow-up, breastfeeding status, number of under-5 children; and exposure to media.

#### Operational definition

*Good hygienic practice during complementary feeding:* The study participants were asked a total of 16 questions: 6 questions related to hand washing with water and soap (using a three-point scale: 1 for “always,” 2 for “sometimes,” and 3 for “wash only with water”) and 10 questions related to safety measures during food preparation (with “Yes” or “No” responses). For the six hand-washing-related questions, responses were dichotomized as 1 for “always” and 0 for “sometimes” or “wash only with water.” Similarly, responses to the 10 food safety questions were dichotomized as 1 for “correct” and 0 for “incorrect.” Participants who answered correctly on more than 55% of the questions were categorized as having good hygienic practices during complementary feeding, while those with fewer correct responses were categorized as having poor hygienic practices (3, 6).

*Knowledge about hygienic complementary feeding:* To assess their knowledge of key moments for hygienic complementary feeding, mothers were asked a question with six correct answers. Each participant received a score reflecting the number of correct answers they provided. Mothers who scored below the average were categorized as having inadequate/poor knowledge, while those who scored at or above the average were regarded as possessing good knowledge of hygienic complementary feeding (3, 6).

*Improved latrines:* Improved latrines include flush toilets connected to a piped sewer system, septic tank, or pit latrine; ventilated improved pit (VIP) latrine, composting toilet, or pit latrine with a slab. Unimproved latrines include pit latrines without slabs, hanging latrines, and bucket latrines (3, 6, 7).

*Improved water sources:* These include piped water, boreholes or tube wells, protected dug wells, protected springs, and packaged or delivered water. Unimproved water sources include unprotected wells, unprotected springs, and surface water (3, 6, 7).

#### Frequency of hand washing with soap

Always was the answer for the mother/caregiver who washes her hands with soap every time she cares for the child, sometimes was the answer for the mother/caregiver who washes her hands with soap occasionally but not every time she cares for the child, and never was the answer for the mother/caregiver who does not wash her hands with soap at any time when caring for the child (14).

*Exposure to media:* A respondent at least reads a newspaper, watches television, or listens to the radio once a week (3, 6, 7).

#### Data collection and quality control

Data were collected through interviews and observations using a structured questionnaire. The questionnaire was meticulously prepared by reviewing previous studies on the hygienic practices of mothers with children aged 6–24 months during complementary feeding. Initially developed in English, the questionnaire was translated into Afan Oromo (the local language) and subsequently back-translated to English to ensure consistency. For the data collection process, five health extension workers (HEWs) were selected as data collectors, and two individuals with Bachelor of Science (BSc) degrees—one in environmental health and the other in public health—served as supervisors. A pretest was conducted on 5% of the sample size (25 participants) in a kebele of Borecha Woreda where the main study was not undertaken. Training was provided to the data collectors and supervisors on the aim of the study, selection criteria, data collection techniques, and how to clarify any ambiguities in the questionnaire before the actual data collection.

Each day after data collection, the questionnaires were reviewed by the supervisors and the principal investigator to ensure completeness. The completeness of the questionnaire was checked before data entry. Any incomplete questionnaires were discarded from the analysis.

#### Data processing and analysis

Data were entered into Epi-Data version 4.6 software and then exported to SPSS statistical package version 20.0 for further analysis. Descriptive statistics were used to present the data. Bivariate and multivariable logistic regression analyses were used to identify factors associated with hygienic complementary feeding practices. In bivariate logistic regression, *p*-value <0.25 was used to retain variables for the multivariable logistic regression model. The Hosmer and Lemeshow test was used to assess the model's fitness to conduct logistic regression. The test gave a *p*-value of 0.79, indicating a good fit.

A backward stepwise variable selection method was used during multivariable logistic regression to control the confounding effect. Crude and adjusted odds ratios (AORs) with 95% confidence intervals (CIs) were computed to assess the association between independent predictors and outcome variables. Multicollinearity was detected by using a metric known as the variance inflation factor (VIF), which measures the correlation and strength of correlation between predictor variables in the regression model. A *p*-value <0.05 was considered statistically significant. The strength of statically significant variables was measured by the values of the odd ratios.

#### Ethical approval and informed consent

An ethical clearance letter was obtained from the Research and Ethical Review Committee of the College of Medicine and Health Sciences at Mettu University, specifically designated for the Borecha Woreda Health Office. Permission was then granted by the Woreda administrative office, and a permission letter was obtained from and for each kebele. Following the acquisition of permission, the issues of confidentiality was explained to each participant. Participants were apprised of the study's purpose and objectives and their rights to withdraw or decline participation, and verbal consent was obtained from all study participants. The confidentiality of the collected data was guaranteed, and individual privacy was upheld.

## Results

### Sociodemographic characteristics of participants

A total of 539 mothers or caregivers with children aged 6–24 months were interviewed for this study. However, two participants declined to participate, and one incomplete response was excluded, resulting in a 99.4% response rate. The majority of participants, 501 (93.5%), were of Oromo ethnicity. In terms of religion, nearly three-fourths, 414 (77.2%), were Muslim. In addition, the majority of the mothers, 504 (94%), were married (Table 2).

**Table 2:** Sociodemographic characteristics of participants in Borecha Woreda, southwestern Ethiopia, May 2022 (*N* = 536).

Variables	Categories	Frequency	Percentage
Age of the respondent (years)	<25	153	28.5
	25–29	198	36.9
	≥30	185	34.5
Religion	Muslim	414	77.2
	Orthodox	71	13.2
	protestant	51	9.5
Ethnicity	Oromo	501	93.5
	Amara	19	3.5
	Others	16	3.0
Marital status of the mother	Married	504	94
	Divorced	24	4.5
	Others	8	1.5
Educational status of the mother	Unable to read and write	349	65.1
	Able to read and write	131	24.4
	Primary school	36	6.7
	Secondary school and above	20	3.7
Occupational status of the mother	Housewife	469	87.5
	Merchant	32	6
	Farmer	11	2.1
	Civil servant	10	1.9
	Others	14	2.6
Household monthly income	Below mean	361	67.35
	Mean and above	175	32.64
Family size	Less than 5	268	50
	5 and above	268	50
Number of under-2 children	One	526	98.1
	Two	10	1.9
Husband’s educational status	Unable to read and write	216	40.3
	Read and write only	181	33.8
	Primary level	45	8.4
	Secondary level	40	7.5
	Diploma and above	32	6
Occupational status of the father	Farmer	350	65.3
	Merchant	118	22.0
	Civil servant	34	6.3
	Others	13	2.4
Place of residence	Rural	423	78.9
	Urban	113	21.1
Access to media (TV or radio)	Yes	293	54.7
	No	243	45.3
Got training on child food preparation	No	474	88.4
	Yes	62	11.6

The average age of the participants was 27.85 years, with a standard deviation (SD) of ±4.698. In addition, 423 (78.9%) of the participants in this study resided in rural areas. Nearly two-thirds, 349 (65.1%), had no formal education. The majority, 469 (87.5%), were housewives by occupation. Half of the households, 268 (50%), had a family size of five or more members. Furthermore, 526 (98.1%) of the mothers had at least one child aged between 6 and 24 months (Table 2).

### Housing and environmental characteristics

Out of 536 respondents, 482 (84.3%) had some type of latrine for their household members. The most common type was a pit latrine with a slab, found in 239 (41.8%) households. Nearly all households, 561 (98.1%), sourced their drinking water from protected sources. In addition, 510 (89.2%) of the households had a separate kitchen for food preparation, and 380 (66.4%) used traditional cook stoves for cooking (Table 3).

**Table 3:** Housing and environmental status of mothers/caregivers’ household in Borecha Woreda, southwestern Ethiopia, May 2022 (*N* = 536).

Variables	Categories	Frequency	Percentage
Presence of latrine	Yes	482	84.3
	No	49	9.1
Type of latrine available (*N* = 482)	Pour-flush latrine	5	0.9
	VIP latrine	13	2.4
	Pit latrine with a slab	258	48.1
	Pit latrine without a slab/open pit	212	39.6
	No facilities or bush or field	48	9.0
Presence of a hand-washing facility near the latrine	Yes	65	12.1
	No	427	79.7
Hand washing with soap after visiting toilets	Always	7	1.3
	Sometimes	515	96.1
	Never	14	2.6
Hand washing with soap after cleaning child's bottom	Always	7	1.3
	Sometimes	515	96.1
	Never	14	2.6
Wash child's hands with soap after he/she defecates	Always	8	1.5
	Sometimes	515	96.1
	Never	13	2.4
Source of drinking water	Piped water	337	62.9
	Protected well water	161	30
	Protected spring water	4	0.7
	Unprotected well water	33	6.2
	Unprotected spring water	1	0.2
Distance to the water source	Water source in the yard	74	13.8
	Less than 30 min	457	85.3
	Greater than 30 min	5	0.9
Household water treatment	Chlorine	98	18.3
	Wuhan agar	2	0.4
	No usage of treatment	436	81.3
Presence of a separate kitchen	Yes	516	96.3
	No	20	3.7
Presence of a separate area to store raw and cooked foods	Yes	531	99.1
	No	5	0.9
Presence of a three-compartment dishwashing facility	Yes	10	1.9
	No	526	98.1
Hand-washing facility near the latrine	Yes	65	12.
	No	471	87.9
Separate kitchen for food preparation	Yes	501	93.5
	No	35	6.5

### Prevalence of hygienic practices during complementary feeding

Among the 536 respondents, 350 (65.3%) of the study mothers/caregivers had poor hygienic practices during complementary feeding. On the other hand, 70 (13.1%) of the participants demonstrated commendable knowledge, and 214 (39.9%) exhibited a positive attitude toward hygienic practices in complementary feeding (Table 4).

**Table 4:** Knowledge, attitude, and practice of the mothers/caregivers during hygienic complementary feeding among children 6–24 months in Borecha Woreda, southwestern Ethiopia, 2022 (*N* = 536).

Variable	Frequency (*n*)	Percentage
Practice of the mothers/caregivers during children’s hygienic complementary feeding		
Good hygienic feeding practices	186	34.7
Poor hygienic feeding practices	350	65.3
Attitude toward hygiene practices (*N* = 536)		
Negative	214	39.9
Positive	322	60.1
Knowledge toward hygiene practices (*N* = 536)		
Poor	468	86.9
Good	70	13.1

### Factors associated with hygienic practice care of the respondents during complementary feeding

In the bivariate analysis, several variables, such as the maternal age, educational attainment of the father or husband, availability of hand-washing facilities near the latrine, maternal knowledge of hygienic practices, source of potable water, and educational level of the mother, demonstrated a significant association with inadequate hygiene practices (*p*-value < 0.05). In the final multivariable logistic regression analysis, maternal age, husband's educational level, and maternal knowledge of hygienic practices were independent risk factors associated with poor hygienic practice during complementary feeding of children aged 6–24 months.

The likelihood of poor hygienic practices among women above 30 years was approximately twofold (AOR: 1.8; 95% CI: 1.11–2.90) greater than that observed in women under the age of 25 years. A mother whose spouse has attained at least a primary level or higher education experiences a 50% reduction in the odds (AOR: 0.50; 95% CI: 0.31–0.83) of engaging in poor hygiene practices during the complementary feeding period compared to a mother whose spouse is illiterate. The likelihood of poor hygiene practices during complementary feeding among mothers with inadequate knowledge of hygienic care was determined to be 2.71 times (AOR: 2.71; 95% CI: 1.60–4.61) higher than that of mothers with adequate knowledge (Table 5).

**Table 5:** Binary logistic regression analysis of factors associated with poor hygienic practices during complementary feeding among women with children in Borecha Woreda, Ethiopia, 2022.

Variables	Poor practice, *N* (%)	Good practice, *N* (%)	Crude odd ratio (95% CI)	Adjusted odd ratio (95% CI)
Age of the mother				
<25	96 (17.91)	57 (10.63)	1	1
25–29	114 (21.27)	84 (15.67)	0.81 (0.52–1.24)	0.75 (0.48–1.17)
≥30	140 (26.12)	45 (8.40)	1.85 (1.16–2.96)	1.80 (1.11–2.92)
Educational status of the husband				
Unable to read and write	158 (29.48)	64 (11.94)	1	1
Can read and write	122 (22.76)	64 (11.94)	0.77 (0.51–1.17)	0.77 (0.50–1.18
Primary and higher	70 (13.06)	58 (10.82)	0.49 (0.31–0.77)	0.50 (0.31–0.83)
Hand-washing facility near the latrine				
No	316 (58.96)	155 (28.92)	1.86 (1.10–3.14)	1.45 (0.81–2.58)
Yes	34 (6.34)	31 (5.78)	1	1
Knowledge of hygiene practices				
Poor	316 (58.96)	150 (27.99)	2.23 (1.34–3.70)	2.71 (1.60–4.61)
Good	34 (6.34)	36 (6.72)	1	1

## Discussion

This research endeavor sought to evaluate hygienic practices during the complementary feeding process and the related factors among mothers of children aged 6–24 months in Borecha Woreda, located in the southwestern region of Ethiopia. According to the findings of this investigation, the prevalence of poor complementary food preparation practices among mothers was determined to be 65.30%. Variables such as the maternal age, the educational background of their husbands, and the women's knowledge regarding hygiene practices were found to be correlated with substandard hygienic practices in maternal complementary feeding.

The results of our investigation indicated that the incidence of inadequate hygienic practices in the preparation of complementary foods was 65.3% (95% CI: 61.3%–69.0%). This observation aligns with the findings from a study conducted in the Harari region of Ethiopia (61%) (15) and other studies in Ethiopia (16), as well as those from Tegedie Woreda in northwest Ethiopia (66%) (3) and Bahir Dar Zuria Woreda in northwest Ethiopia (61.1%) (6). A plausible explanation for this congruence may be the socioeconomic similarities or comparable living standards of the populations involved.

In this study, the impact of maternal age on the hygienic preparation and feeding practices of complementary foods for children was identified. Mothers aged over 30 years exhibit approximately a twofold increased likelihood of engaging in poor hygienic practices during the complementary feeding period compared to their counterparts who were under 25 years of age. This observation aligns with findings from a study conducted in Bahir Dar Zuria Woreda, Ethiopia (6). A possible explanation for this phenomenon may be the growing neglect of hygienic practices in preparing complementary foods and feeding children as the mother's age advances. Conversely, this finding contrasts with the results of a study conducted in the Harari region (15). The observed discrepancies may be attributable to socioeconomic variables or potential errors in the study, given the variations in sample sizes utilized.

According to the findings of this study, the educational level of the husband is a significant factor that affects hygienic practices during the complementary feeding process. Women whose husbands have completed primary education or higher demonstrate a 50% greater likelihood of engaging in appropriate hygienic practices during complementary feeding compared to those whose husbands lack basic literacy skills. This phenomenon may be attributed to the tendency of educated husbands to guide mothers regarding the safe preparation, handling, and storage of food, as well as the proper disposal of child feces and appropriate feeding methodologies. In addition, educated fathers may disseminate knowledge concerning the adverse effects of unsanitary practices on the health of their children. Empirical evidence from studies conducted in Ethiopia, Harari, and Tanzania indicates that mothers with higher levels of education practice better hygiene during the preparation and handling of complementary foods (15, 17, 18, 19). Furthermore, the educational background of the fathers, or the collective education of both parents, may also influence the family's economic circumstances. For instance, fathers with advanced educational qualifications are more likely to secure higher-paying employment, which can result in enhanced living conditions and greater access to hygienic facilities (e.g., pit latrines, water treatment systems). In this context, parental education, along with environmental factors and infrastructural conditions such as access to clean water and adequate sanitation facilities, may substantially influence the hygienic care practices adopted for children.

Inadequate knowledge of social protection programs and their relation to feeding practices, as well as limited decision-making power over money and assets, may affect the hygienic care practices of the mother (14). The findings from our study indicated that mothers with poor knowledge of hygienic food handling were nearly three times more likely to engage in poor hygienic practices during complementary feeding compared to knowledgeable mothers. This is in agreement with studies conducted in Bale Zone, southeast Ethiopia (18) and Debarq, northwest Ethiopia (19). A possible reason could be that mothers with a good level of knowledge about hygienic food handling are more aware of the consequences of poor food handling practices and practice better hygiene compared with those with poor knowledge of complementary food safety. A good level of knowledge among participants might enable and influence participants’ attitudes and practices toward food safety.

### Limitations and strengths of the study

This investigation was carried out at the community level utilizing an appropriate sampling method; thus, the findings of the study may serve as a more accurate representation of the target population. Nonetheless, the study has its limitations. Given that both exposure and outcome were evaluated simultaneously, there could have been a temporal bias. In addition, social desirability bias may have been introduced during the interviews with study participants. Moreover, the lack of universally recognized knowledge categories required dependence on existing evidence. Our flexible criteria for “good” knowledge might lead to an underestimation of its prevalence. Our findings were influenced by previous literature.

## Conclusion and recommendations

### Conclusion

The prevalence of inadequate hygiene practices in the preparation and handling of complementary foods by mothers of children aged 6–24 months in Borecha Woreda was high compared to that reported in the existing literature. Moreover, the mother's age, the husband's educational level, and the mother's knowledge of hygienic food handling were associated with poor hygienic practices during complementary feeding.

### Recommendations

Based on the results of this study, there is a pressing need for strategies and policies aimed at enhancing the knowledge of mothers and husbands regarding hygienic practices in complementary feeding. This can be achieved through community health education, training, or awareness programs for breastfeeding mothers and families, emphasizing the importance of hygienic care during complementary feeding. Consequently, the Borecha Woreda Health Office should recognize the significant need for extensive orientation, comprehensive awareness campaigns, and trust-building initiatives for women responsible for child feeding, as well as for the community at large.

For researchers interested in this field, we recommend that it would be beneficial to incorporate qualitative methods such as focus group discussions alongside quantitative studies. In addition, conducting thorough, long-term prospective observational studies may yield more substantial evidence on hygienic practices during complementary feeding. Researchers aiming to understand the behavioral differences among Ethiopian millennials should consider conducting targeted studies. These investigations could investigate policy changes, hygiene education efforts, and sociocultural factors that might impact behavior.

## Data Availability

The raw data supporting the conclusions of this article will be made available by the authors without undue reservation.
